# The expression profile of human peripheral blood mononuclear cell miRNA is altered by antibody-dependent enhancement of infection with dengue virus serotype 3

**DOI:** 10.1186/s12985-018-0963-1

**Published:** 2018-03-22

**Authors:** Liming Jiang, Qiangming Sun

**Affiliations:** 1 Institute of Medical Biology, Chinese Academy of Medical Sciences, and Peking Union Medical College, 935 Jiao Ling Road, Kunming, Yunnan Province 650118 People’s Republic of China; 2 Yunnan Key Laboratory of Vaccine Research & Development on Severe Infectious Diseases, Kunming, 650118 People’s Republic of China

**Keywords:** PBMC, DENV, miRNA, Antibody dependent enhancement

## Abstract

**Background:**

Antibody-dependent enhancement (ADE) of dengue virus (DENV) infection has been identified as the main risk factor for severe dengue disease, although the underlying mechanisms leading to severe dengue fever remain unclear. MicroRNAs (miRNAs) participate in numerous pathological and biological processes, including host responses to viral infections.

**Method:**

Here, we aimed to investigate the differences in miRNA expression patterns in human peripheral blood mononuclear cells (PBMCs) infected with DENV-3 and DENV-3-ADE at various time points employing high-throughput sequencing.

**Results:**

According to miRNAs high-throughput sequencing, a total of 50 known miRNAs exhibited significant differences. GO (Gene Ontology) and pathway analysis of the predicted targets showed enrichment in the regulation of transcription, including multicellular organismal development, DNA-dependent transcription, negative regulation of cell differentiation and transcription. Afterwards, regulatory networks of miRNA predicted targets, miRNA transcription factors, miRNA pathways and miRNA GOs were formulated to expose the complex regulatory mechanisms of miRNAs during the infection phase. Finally, we analyzed hierarchical GO categories of the predicted targets involved in the MAPK signaling pathway, the cGMP-PKG signaling pathway, the cAMP signaling pathway, the endocytosis effect, and our analyses indicated that innate and adaptive immunity following DENV-3 and DENV-3-ADE infections may be signally distinct.

**Conclusion:**

Our results demonstrate a novel describing miRNA expression profiles in human PBMCs with DENV-3 and DENV-3-ADE infections using high-throughput sequencing. Our findings could provide a beneficial basis for further studies on the regulatory roles of miRNAs relevant to the different immune responses caused by DENV-3 and DENV-3-ADE infections.

**Electronic supplementary material:**

The online version of this article (10.1186/s12985-018-0963-1) contains supplementary material, which is available to authorized users.

## Background

Dengue virus (DENV) is a mosquito-borne virus that causes serious widespread public health issues in tropic and sub-tropical areas of the world. There are more than 100 countries where DENV is endemic, and the hardest hit areas are in Asia and Latin America. More than 50 million people are infected with dengue virus each year [1]. DENV belongs to the *Flavivirus* genus and is transmitted by *Aedes albopictus* and *Ae. aegypti* mosquitos. Global warming and geographic expansion of the vector contributes to a continuous increase in the incidence and severity of the disease [2, 3]. There are four distinct serotypes of DENV (DEVN I–IV), and each of them can cause a spectrum of symptoms from subclinical to hemorrhagic fever and death [4]. Frequently, preexisting heterotypic sub-neutralizing antibodies of dengue virus have been shown to contribute to the pathogenesis of severe dengue, that result from antibody-dependent enhancement (ADE) [5, 6]. Partial severe dengue fever is due to the ADE of DENV infection, and probably via the interaction between virus/antibody complex and Fc receptors on cell surface. The secondary heterotypic infection or waning immunity of infants born to mothers infected with DENV has been shown to potentiate secondary infection of monocytes and macrophages via ADE [7, 8].

In vitro experiments have shown that enhanced infection of Fc-receptor bearing cells, such as THP-1 and K562, resemble those of DHF/DSS patients [9–11]. Thus, human antibodies are believed to play complicated roles in controlling DENV infection. It is important to distinguish antibodies with neutralizing or enhancing activities against DENV for both basic and applied research.

Clinical observation indicate that elevated viraemia is normally accompanied by a high concentration of immunomodulatory and proinflammatory cytokines [12]. Chareonsirisuthigul T using the THP-1 cell line found that DENV-ADE infection could suppress the expression of TNF-α, IFN-γand IL-12, while stimulating the expression of the anti-inflammatory cytokines IL-10 and IL-6 [13]. Anti-inflammatory cytokines play an important role in IFN antiviral pathway, particularly IL-10 [14].

MicroRNA is a class of small noncoding RNAs that are cellularly and endogenously encoded single-stranded RNAs of approximately 22 nucleotides in length. They are associated with and contribute to several cellular functions, such as apoptosis, differentiation, development, and they act as key regulators of gene expression at the post-transcriptional level by targeting messenger RNAs (mRNAs) for degradation or translational repression [15, 16]. RNA polymerase transcribes the pri-miRNA to miRNAs [17]. In addition, abnormal expressions of miRNAs results in dysregulated innate and adaptive immunity, which can cause autoimmune diseases and hematopoietic malignancies [18, 19]. Moreover, accumulating research has indicated that virally encoded miRNAs can regulate viral or cellular gene expression and therefore contribute to replication and viral pathogenesis [20, 21]. Thus, depicting the emerging roles of cellular and virus-encoded miRNAs in host-pathogen interactions may have huge significance in the development of new antiviral therapies and the manipulation of regulatory molecules. Plentiful studies have determined that DENV infection can influence host miRNA expression profiles and that changes in these profiles are involved in immune escape and antiviral responses in DENV infection [22].

Hence, we hypothesized that the host miRNAs induced by DENV infection might be distinct from those that are induced by DENV-ADE infection. PBMCs, consisting of lymphocytes (NK cells, B cells and T cells), dendritic and monocytes cells, are thought to be an essential component of the immune system, and alterations in PBMC populations are most likely linked to the clinical features that occur during the progression of viral infection. Therefore, we utilized high-throughput sequencing of miRNA expression profiles in human PBMCs infected with DENV and DENV-ADE to investigate why these viruses result in diverse immune responses and clinical features. The results from this study could provide new perspectives regarding the mechanisms underlying DENV and DENV-ADE pathogenesis.

## Methods

### Cell culture and virus infection

PBMCs were separated from EDTA anticoagulated whole blood samples from healthy adult males with no acknowledged diseases or infections and were isolated by Ficoll-Hypaque gradient centrifugation as adapted in the experimental operating instructions. The level of DENV IgG antibodies detection of donors serum were commissioned by Xishuangbanna Dai Autonomous Prefecture People’s Hospital, the test results showed that the DENV IgG antibodies of donors was negative. Stated, PBMCs plated in T25 flasks at 4.2 × 10^5^ cells per ml were cultivated in RPMI 1640 medium (BI, China) supplemented with 10% fetal bovine serum (FBS, SJQ, China) plus penicillin and streptomycin and incubated 2 h at 37 °C in 5% CO_2_ in a humidified incubator. Two hours later, the DENV-3 virus strainthat originated from an epidemic in Guangdong, China in 2014 and DENV-2 anti-prM monoclonal antibody (abcam, AB41473) were added in PBMCs at a multiplicity of infection (MOI) of 5.Cells were infected in triplicate and collected at 0, 8 and 24 h post infection (hpi). Cells infected with DENV-3 and DENV-3 plus DENV-2 anti-prM complex at 0 hpi were used as controls. We defined the different experimental groups as DENV-3–0 h, DENV-3–8 h, DENV-3–24 h, DENV-3-ADE–0 h, DENV-3-ADE–8 h and DENV-3-ADE–24 h. Additionally, a subset of the DENV-3-0 h and DENV-3 ADE–0 h groups were subjected to normalization (the normalization value was set to 1), and these two groups were then designated Con.

### miRNA extraction from PBMCs and quality control

miRNA was isolated from cultured DENV-3 and DENV-3-ADE infected PBMCs according to standard miRNeasy Mini Kit (Agilent technologies Santa Clara, US)protocols. The integrity and quality of RNA were evaluated using RNA 6000 Nano Lab Chips on an Agilent 2100 Bioanalyzer (Agilent technologies Santa Clara, US) and estimated by reviewing electropherograms and the RNA integrity number (RIN) of each sample (Table 1). Qualified miRNA samples from three independent experiments of each group were pooled and used for subsequent deep sequencing and library construction.

**Table 1:** Details of small-RNA sequencing information and subsequent data analysis

Sample Name	Raw Reads	Effective reads	Q20 Value	Result
0	22,570,354	20,008,695	98.26%	Pass
DENV3–8	20,911,454	17,884,645	98.46%	Pass
DENV3–24	21,642,821	18,414,136	98.24%	Pass
ADE8	20,921,471	19,445,109	98.41%	Pass
ADE24	21,220,416	20.552.976	98.26%	Pass

Raw Reads: the numbers of raw sequencing data read; Effective reads: the numbers of Raw Reads that passed both quality filtering steps; Q20 = bases of Q > =20 / all bases of sequencing

### Micro RNA (miRNA) library construction, sequencing and analysis

High-throughput sequencing technology (transcriptome analysis tool), not only detects known transcripts but also promote the discovery of novel transcripts [23]. MicroRNA library construction and sequencing was accomplish by the National Engineering Center for Biochip in Shanghai on an Illumina HiSeq 2000 system.The sequencing data were submitted to the Gene Expression Omnibus (GEO) database (www.ncbi.nlm.nih.gov/geo/) under the accession number GSE98859.

### Bioinformatic analysis of sequencing data

#### Prepare of high-throughput sequencing technology analysis

miRNAs with a *P* value < 0.05 and a fold change ≥2 or ≤ 0.5 were treated significantly different among the groups. We first characterized differentially expressed miRNAs using log2-fold changes in the ratios of the detected signals [log2(infected/control)]. To isolate pivotal differences between the DENV-3- and DENV-3-ADE-infected samples, we identified a set of unique expression patterns in accordance with different signal density changes in miRNAs in different situations.The potential targets of the differentially expressed miRNAs were predicted with two miRNA target prediction algorithms: miRDB and TargetScan [24, 25].The parameters for miRDB and TargetScan were set as the top 50 genes and target score ≥ 99, respectively.

#### Gene ontologyKEGGpathway analysis

To sytemically describe the property and function of target genes and their products, the Protein Analysis THrough Evolutionary Relationships (PANTHER) classification system version 9.0 was used to classify genes and proteins to expedite high-throughput analysis.

#### Regulatory network analysis

In viewof gene networks can clearly show the interactions betweenmiRNAs and their target genes, and the biological processes that are mediated by these target genes. Thus, miRNA-gene network was constructed in this study based on the targeted regulatory relationships between miRNAs and their target genes. Target genes associated with cell differentiation, cellular antiviral immune response, signal transduction, response to stimulus, regulation of molecularfunction, apoptosis etc. were selected based on the annotations of enriched KEGG and GO pathway terms. The regulatory networks of core regulatory miRNAs and their target genes were depicted by the software Cytoscape. Thenceforth, based on interactions between key differentially expressed miRNAs and their targets, GOs and target genes, pathways and targets, regulatory networks for miRNAs, including (a miRNA-pathways network, miRNA-targets network and miRNA-GOs network) were created.

### Quantitation of gene expression levels by RT-qPCR

RT-qPCR was used to investigate the relative levels of gene expression among DENV nad ADE infection PBMCs during 48–96 h. Briefly, PBMCs cellular RNA was extracted using standard miRNeasy Mini Kit (Agilent technologies Santa Clara, US)protocolsand then subjected to reverse transcription with a first-strand cDNA synthesis kit before amplification by RT-qPCR. The amplifications were performed using SYBR Advantage qPCR Premix ((TIANGEN, CHINA) with a Bio-Rad CFX96 detection instrument.

### Validation of miRNAs and miRNA target genes by RT-qPCR

The quantitative reverse transcription-polymerase chain reaction was used to validate miRNA expression. For further confirmation, we randomly selected 6 differentially expressed miRNAs for RT-qPCR analysis. miRNA expression was tested by poly(A)-tailed RT-qPCR. For each sample, 1μg of total RNA was polyadenylated and reverse transcribed using poly(A)polymerase with a miScript II RT Kit (QIAGEN, USA), in accordance with the manufacturer’s instructions. Subsequently, each cDNA was amplified on a 7900 HT Sequence Detection System (ABI, USA) with an mRQ 3primer and miRNA-specific 5 primers to quantify specific miRNA sequences; the amplifications were performed using SYBR Advantage qPCR Premix ((TIANGEN, CHINA). All miRNA-specific 5 primers used in the qPCR experiments are shown in the Additional files.

### Statistical analysis

For sequencing data, raw reads achieved from each library were normalized to TPM. For RT-qPCR, the data are expressed as the mean ± standard error of the mean (SEM). Statistical analysis was measured using STATA 11.0 software (stata Corp, College Station, TX, USA). P - value of less than 0.05 was considered to indicated a statistically significant difference.

## Results

### Global analysis of miRNA expression patterns in response to direct infection and ADE infection with DENV-3

To determine the changes in miRNA expression patterns in PBMCs during direct infection and ADE infection with DENV-3, global cellular miRNA expression patterns following infection were compared with those of the controls. In this study, only those differentially expressed miRNAs with a *p* value < 0.05 and a fold change ≥2 or ≤ 0.5 are described. The results showed that, compared with the DENV-3–0 h group, there were 8 up-regulated and 7 down-regulated known miRNAs in the DENV-3–8 h group, as well as 13 up-regulated and 1 down-regulated known miRNAs and 1 up-regulated novel miRNA in the DENV-3–24 h group. Moreover, there were 10 up-regulated and 1 down-regulated known miRNAs in the ADE infection–8 h group, as well as 37 up-regulated and 4 down-regulated known miRNAs in the ADE infection–24 h group relative to the ADE infection-0 h group (Additional file 1: Table S1, Additional file 2: Figure S2, Table 2).

**Table 2:** Significantly differentially expressed miRNAs during ADE and direct infection

Comparison	Known differentially expressed miRNAs			Novel differentially expressed miRNAs		
	Total	Up	Down	Total	Up	Down
DENV3–8 vs. Mock	15	8	7	0	0	0
DENV 3–24 vs. Mock	14	13	1	1	1	0
ADE-8 vs. Mock	11	10	1	0	0	0
ADE-24 vs. Mock	41	37	4	0	0	0

Mock: Blank culture of PBMCs cells

**Fig. 1:**
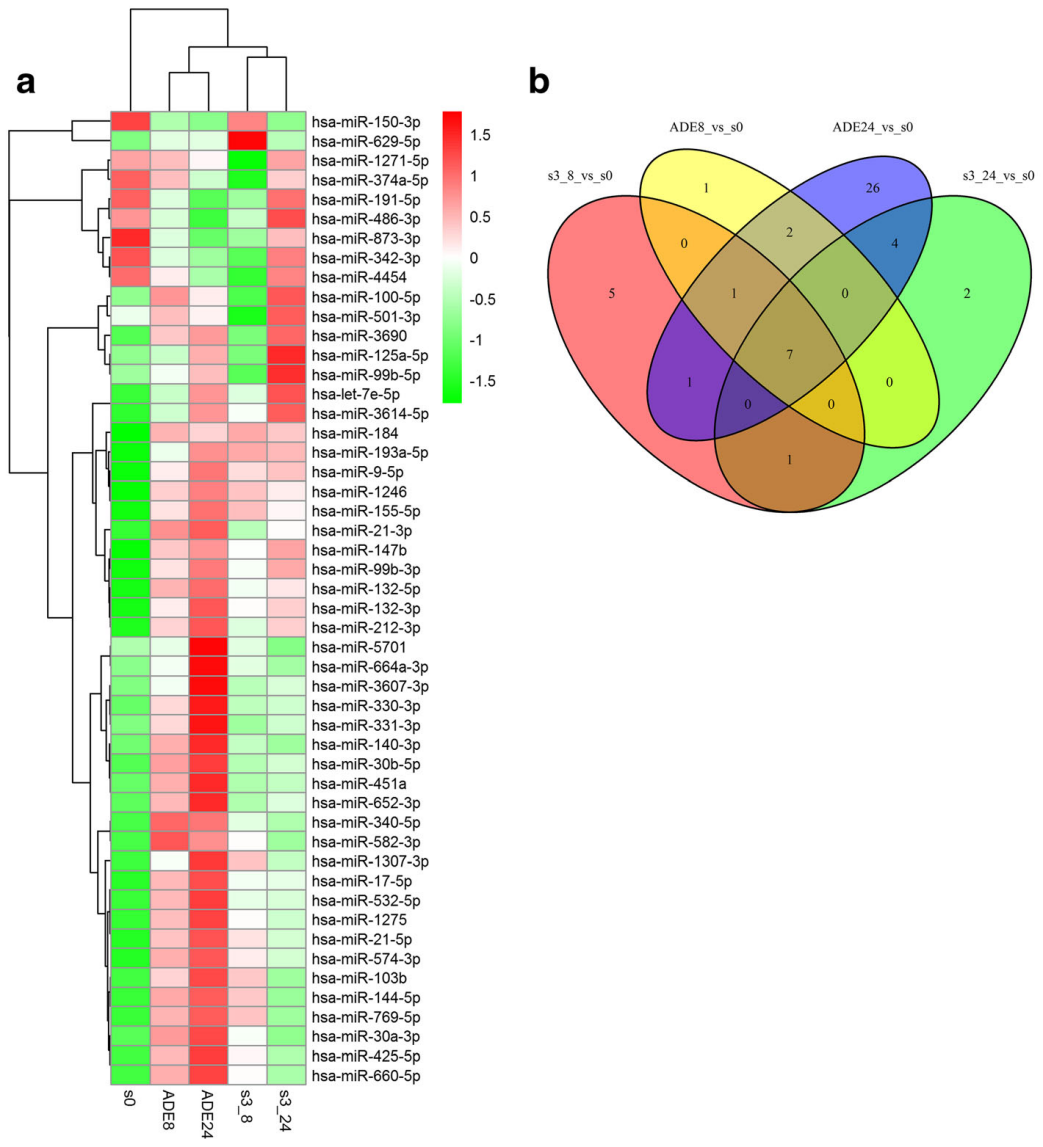
Global analysis of differentially expressed miRNAs. **a** Hierarchical cluster of differentially expressed miRNAs during the DENV-3 and ADE infections at 8 h and 24 h relative to control samples. Significance was determined using a fold-change threshold of at least 2 and a *P* value cutoff of 0.05, numbers with s0 denote normal controls. **b** Venn diagram of all differentially expressed miRNAs in all samples. Different colors represent different experimental groups as indicated and the numbers 1, 2, 4, 5, 7 and 26 show the differentially expressed miRNAs that were significantly changed during infection with DENV-3 and ADE

Subsequently, the sum aggregates of differentially expressed known miRNAs were taken from four groups. Then, we used the R language heatmap package to draw an miRNA Heatmap diagram; 50 differentially expressed miRNAs were used to perform hierarchical clustering to construct a heat map based on differential expression patterns with log 2 values (infected/control) and fold changes (Fig. 1a). A positive log 2 value indicated up-regulation, and a negative log 2 value indicated down-regulation. The data for all the differentially expressed miRNAs were also graphed as a Venn diagram (Fig. 1b). Common and distinct differentially expressed known microRNAs in response to direct infection and ADE infection with DENV-3 across all time points were revealed. We found that the patterns induced by DENV-3 differed from those induced by ADE infection at 8 h and 24 h. In addition, the miRNA expression patterns induced by DENV-3 at 8 h also differed from those induced by DENV-3 at 24 h, and the same was true for ADE infection. Therefore, these results demonstrated that miRNA expression patterns in PBMCs following infection with DENV-3 and ADE of DENV-3 are mode-and time-specific and the ADE infection at 24 h group differed the most from the other three groups.

To further confirm our sequencing data, six significantly differentially expressed miRNAs, including hsa-miR-184, hsa-let-7e-5p, hsa-miR-132-3p, hsa-miR-155-5p, and hsa-miR-1246, were chosen for RT-qPCR analysis. The results showed a general consistency between RT-qPCR and high-throughput sequencing results (Additional file 3: Figure S1).

### Prediction and annotation of miRNA putative target genes

**Fig. 2:**
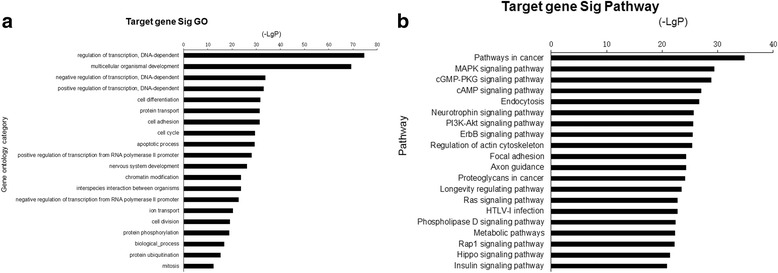
**a**, Analysis of GO functional enrichment of target genes of differentially expressed miRNA during infection between DENV-3 and DENV-3-ADE. **b**, Pathways predicted to be induced by differentially expressed miRNA during the DENV-3 infection. Enrichment score is presented as the negative log_10_ of the P value (-log_10_P) and is plotted on the x-axis

To explore the possible regulatory roles of the 50 known differentially expressed miRNAs during DENV-3ADE and direct infection, we used the TargetScan and miRDB databases to predict possible targets genes. There were 12,509 genes found with TargetScan and 129,810 potential genes found with miRDB. Then, the genes that were identified by both programs were selected as the final target genes for subsequent GO and pathway analysis, and 6494 target genes were screened out (Additional file 4: Table S2). Seventy GO terms were enriched based on a *P* value < 0.0001 and FDR < 0.0001. The results are shown in Additional file 5: Table S3. The most obvious GO terms for the miRNA targets included DNA-dependent regulation of transcription, multi-cellular organism development, DNA-dependent negative regulation of transcription, DNA-dependent positive regulation of transcription, cell differentiation, protein transport, cell adhesion, cell cycle, apoptotic process, positive regulation of transcription from the RNA polymerase II promoter, nervous system development, chromatin modification, interspecies interaction between organisms, negative regulation of transcription from the RNA polymerase II promoter, ion transport, cell division, protein phosphorylation, biological-process, protein ubiquitination and mitosis (Fig. 2a). Then, the predicted targets genes were subjected to KEGG pathway enrichment analysis using the PANTHER Classification System. We obtained 122 important pathways based on *P* value <1E^− 7^ and FDR < 1 E^− 07^, and the results are shown in Additional file 6: Table S4. As with the GO analysis We have displayed the notable pathways of the target genes using a histogram (Fig. 2b). The notable pathways included the pathways in cancer, MAPK signaling pathway, the cGMP-PKG signaling pathway, the cAMP signaling pathway, endocytosis, the neurotrophin signaling pathway, the PI3K-Akt signaling pathway, regulation of the actin cytoskeleton, focal adhesion, axon guidance, proteoglycans in cancer, the longevity-regulating pathway, the Ras signaling pathway, HTL-I infection, the phospholipase D signaling pathway, metabolic pathways, the Rap1 signaling pathway, the Hippo signaling pathway, and the insulin signaling pathway. These results showed that numerous biological processes participate in DENV-3ADE and direct infection of PBMCs.

**Fig. 3:**
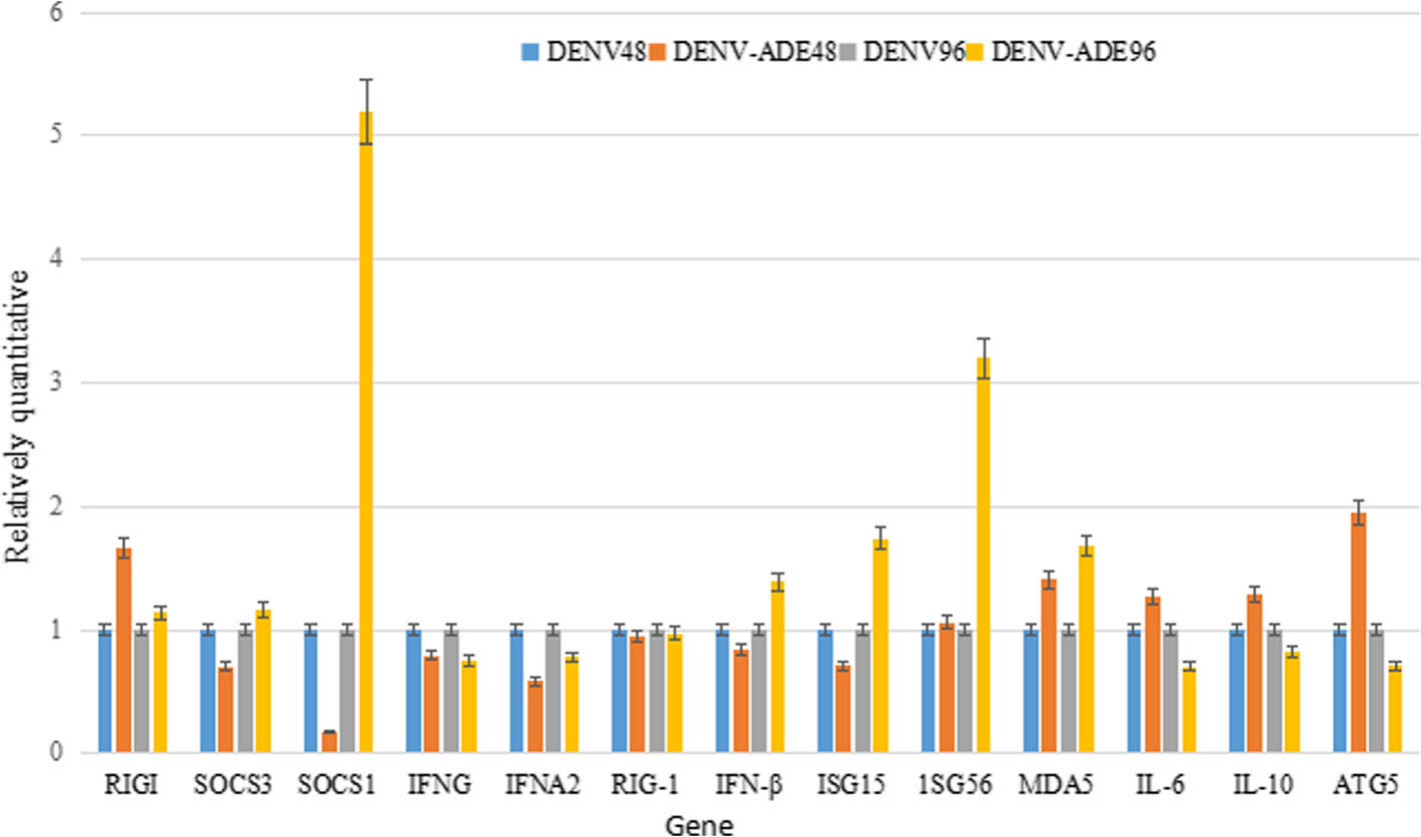
The expression levels of innate immunity- and autophagy-related genes in PBMCs.*, *P* < 0.05; **, *P* < 0.01; ***,*P* < 0.001; #, *P* > 0.05. The levels of innate immunity related genes are the comparison of PBMC infected with DENV to PBMC infected with DENV-ADE

According to the results of miRNA high-throughput sequencing and target gene prediction analysis, autophagy and innate immunity pathways were also targeted by miRNA. In the process of DENV ADE infection, the expression levels of the SOCS1, SOCS3, RIG-1 and ISG56 genes was found to be different (≥2) comparing to the levels during DENV-3 direct infection. In addition, they showed expression trend that were the opposite of their related miRNAs, hsa-miR-330-3P, hsa-miR-342-3P, hsa-miR-501-3P, hsa-miR-331-3P, hsa-miR-340-3P, hsa-miR-3607-3P, hsa-miR-3614-3P and hsa-miR-374a-3P. It was worth noting that the expression levels of innate immunity and autophagy-related genes RIG-I, MDA5 and ATG5 significantly increased 48 h after DENV-ADE infection, while the expression levels of SOCS1, SOCS3, INF-a and ISG15 decreased. Meanwhile, the expression levels of SOCS1, INF-a, ISG15, and ISG56 greatly increased 96 h after DENV-ADE infection (Fig. 3).

### miRNA gene, GO term, and pathway regulation network construction

**Fig. 4:**
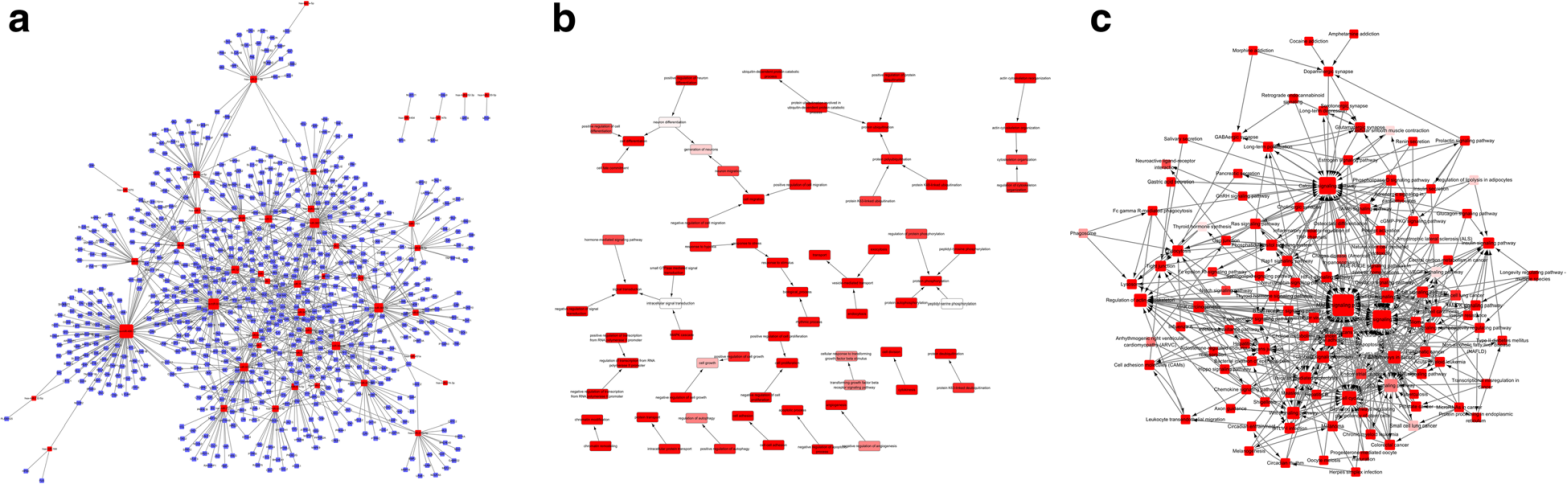
**a**, Regulatory network of miRNAs and their target genes. The red rectangles represent the microRNAs, the blue rectangles represent genes, and the lines represent the regulation relationships between the microRNAs and the genes. **b** The relationship between significant gene functions. Rectangles represent the difference gene functions, and the lines represent the relationships between the functions of the difference genes. Dark to pale red represents small to large *p* values, respectively. **c** The pathway interaction network. In the figure, the squares represent the pathways, and the lines represent the interrelations of the inter-graphs. Dark to pale red represents small to large p values, respectively

To further profile the function of miRNAs, complex network systems were built, including a network of miRNA genes, a network of important GO terms and a network of pathways. Firstly, the intersections of notable target genes in the GO enrichment and KEGG analysis were selected for constructing the regulatory network of miRNA genes (Fig. 4a). The target genes that were most highly regulated by miRNAs were FZD3, SLC38A2, CCNG2, EIF4E, FRS2, MAP3K2, MAPK8, MEF2C, PCGF5, PRKAA2, SCN1A, SCN7A, SEMA6A, SIX4, SMAD2, SMG1, SORT1, SS18, UBE2D1, and UBE2G1. Secondly, we built a network of significant gene functions based on the Gene Ontology database (Fig. 4b), and the results showed that the GO classifications were mainly related to cell division, cell growth, protein ubiquitination, signal transduction, stress response, etc. Furthermore, the pathway regulation network was constructed and is shown in Fig. 4c. According to the interaction network of significant pathways, the main signaling pathways were the MAPK signaling pathway, the PI3K-Akt signaling pathway, the calcium signaling pathway, apoptosis, etc. Of these, the MAPK signaling pathway plays an important role in the whole signal pathway network.

## Discussion

Almost half of the world’s population is at risk for dengue infection. Antibody-dependent enhancement (ADE) has been thought to be involved in the immuno-pathogenesis of severe dengue forms, including dengue hemorrhagic fever (DHF) and dengue shock syndrome (DSS). Currently, CYD-TDV (live-attenuated chimeric yellow-fever-dengue virus tetravalent vaccine) is the only vaccine approved for the prevention of dengue and has now been licensed in 13 countries. However, the safety of this vaccine is a major concern. In clinical trials in Asian countries, antibody-dependent enhancement (ADE), where incomplete vaccination induced priming of seronegative patients and led to subsequent severe disease after natural infection, has become a major safety concern [2, 25–28].Thus, clarifying the pathogenesis of DENV-ADE is important for the development of safe vaccine and therapeutic approaches.

It is known that miRNAs expression affects viral infection in addition to playing a general role in differentiation, apoptosis, cell proliferation, and immunity [29–31]. However, there are few reports on the altered expression levels of miRNAs between DENV-ADE and direct infection of PBMCs, and their precise roles are not clear [32]. In this study, we focused on changes in miRNA expression during DENV-ADE or direct infection of PBMCs to determine the involvement of miRNAs in DENV pathogenesis and the replication cycle and provide insight into vaccine research and therapeutic approaches. A total of 50 known miRNAs were significantly changed in PBMCs following dengue virus direct infection and ADE infection, and of these, both common and unique miRNAs were identified. miRNAs are pivotal gene regulators that act on mRNAs to cause either translation inhibition or mRNA degradation, and they participate in numerous physiological and pathological processes [15, 33].

Hence, it is not surprising that abnormal miRNA expression may result in the pathogenesis of multiple diseases. Growing evidence has indicated that most virus infections alter the expression of cellular miRNA and that cellular miRNAs can modulate viral pathogenesis and replication by regulating the expression of viral or host genes [34, 35]. Several studies have also been conducted to explore the effects of miRNAs on DENV infection [36]. Na et al. found that DENV2 infection significantly decreased the expression of miR-223 in HepG2, EAhy926 and Vero cells, indicating that the down-regulation of miR-223 may be a common event in DENV infection.

Together with these studies, two important points were demonstrated: 1) differential miRNA expression profiles are produced at different time points following dengue virus direct infection and ADE infection, and 2) alterations of common and unique differentially expressed miRNAs following dengue virus direct infection and ADE infection can be used as diagnostic markers and may be therapeutic targets that are worth exploring.

To further elucidate the molecular pathogenesis of dengue virus direct infection and ADE infection, we performed GO and pathway analysis of the potential targets of 50 differentially expressed miRNAs. In this analysis, the observed changes in biological processes (especially related to immune system processes and apoptotic processes) and pathways (especially associated with immune pathways, such as pathways in cancer, the MAPK signaling pathway, the cGMP-PKG signaling pathway, the cAMP signaling pathway, focal adhesion, metabolic pathways and the Rap1 signaling pathway) suggested that the regulation of miRNAs plays an important role in modulating immune responses and the viral pathogenesis of DENV-ADE and direct infection. An increasing number of studies have also verified that miRNAs play critical roles in regulating the immune response, including the proliferation, differentiation, cell fate determination, and function of immune cells as well as in inflammatory mediator release and in modulating intra-cellular signaling pathways [37, 38]. For example, two of the above miRNAs (miRNA-146b-5p and let-7e-5p) have been verified playing an important role in the regulation of the NF-kappaB signaling pathway during viral infections [39], and the NF-kappaB signaling pathway is critical to innate and adaptive immunity and inflammation [40]. It is notable that immune system processes, apoptotic processes, cell proliferation and differentiation were also play a key role in cancer development. Therefore GO and pathway analysis showed that pathways in cancer were the most important during DENV-ADE infection. These findings provide a possible explanation for the pathomechanism that result from dengue virus direct infection and ADE infection. We are very sorry that the control group is insufficient on MOCK-8 and MOCK-24.

The expression of the main potential innate immunity and inflammation signaling molecules regulated by miRNAs, including SOCS1, SOCS3, RIG-1, ISG15, ISG56, IFN-γ, IFN-α, IFN-β, IL-10, IL-6, ATG5, ATG12, RIG-I and MDA-5, have been verified by Q-PCR. Of these molecules, RIG-I, MDA5, ATG5, SOCS1, SOCS3, INF-a, ISG15and ISG56 showed differences in expression levels at different time points after DENV-3 or DENV-3 ADE infection.

## Conclusion

In conclusion, this study compared miRNA expression profiles and related gene regulatory networks between direct and ADE infections of DENV-3 in PBMCs. Our findings could provide a valuable basis for further studies on the regulatory roles of miRNAs related to the different immune and pathological mechanism caused by DENV-3 and DENV-3-ADE infections.

## Additional files

Additional file 1: **Table S1.** The differential expression of miRNAs among DENV-3–0 h, DENV-3–8 h, DENV-3–24 h, DENV-3-ADE–0 h, DENV-3-ADE–8 h and DENV-3-ADE–24 h groups. Including the description of miRNAs and comparison of multiple differences of PBMC infected with DENV to PBMC infected with DENV-ADE. (XLSX 16 kb)

Additional file 2: **Figure S2.** DENV-ADE infection increased virus replication. PBMC cells were infected with DENV3 at a MOI of 5 complexed with 4-fold dilutions of anti-prM mAb. Abscissa expression the dilution multiple of DENV-2 prM antibody, Ordinate representation extracellular DENV genome RNA copy number. (PNG 18 kb)

Additional file 3: **Figure S1.** a. Quantitative real-time PCR verify of hsa-miR-1246 among III-8, III-24 and ADE-24. The levels of hsa-miR-1246 is the comparison of PBMC infected with DENV to PBMC infected with DENV-ADE. b. Quantitative real-time PCR verify of hsa-miR-132-3p among III-8, III-24 and ADE-24. The levels of hsa-miR-132-3p is the comparison of PBMC infected with DENV to PBMC infected with DENV-ADE. c. Quantitative real-time PCR verify of hsa-miR-155-5p among III-8, III-24 and ADE-24. The levels of hsa-miR-155-5p is the comparison of PBMC infected with DENV to PBMC infected with DENV-ADE. d. Quantitative real-time PCR verify of hsa-miR-184 among III-8, III-24 and ADE-24. The levels of hsa-miR-184 is the comparison of PBMC infected with DENV to PBMC infected with DENV-ADE. e. Quantitative real-time PCR verify of hsa-let-7e-5p among III-8, III-24 and ADE-24. The levels of hsa-miR-184 is the comparison of PBMC infected with DENV to PBMC infected with DENV-ADE. (ZIP 34 kb)

Additional file 4: **Table S2.** The gene description, transcript accession, gene symbol and gene ID of experimental screened miRNAs among DENV-3–0 h, DENV-3–8 h, DENV-3–24 h, DENV-3-ADE–0 h, DENV-3-ADE–8 h and DENV-3-ADE–24 h groups. The result of the miRNA difference is by comparison of multiple differences of PBMC infected with DENV to PBMC infected with DENV-ADE. (XLSX 638 kb)

Additional file 5: **Table S3.** The total count, differential count, GO Name and *P* Value of enrichedGO terms among DENV-3–0 h, DENV-3–8 h, DENV-3–24 h, DENV-3-ADE–0 h, DENV-3-ADE–8 h and DENV-3-ADE–24 h groups. The result of the GO difference is by comparison of multiple differences of PBMC infected with DENV to PBMC infected with DENV-ADE. (XLSX 12 kb)

Additional file 6: **Table S4.** The total count, differential count and P Value of enrichment pathway among DENV-3–0 h, DENV-3–8 h, DENV-3–24 h, DENV-3-ADE–0 h, DENV-3-ADE–8 h and DENV-3-ADE–24 h groups. The result of the Path difference is by comparison of multiple differences of PBMC infected with DENV to PBMC infected with DENV-ADE. (XLSX 17 kb)
