# Supplementary figures and images for: The expression profile of human peripheral blood mononuclear cell miRNA is altered by antibody-dependent enhancement of infection with dengue virus serotype 3
Source: Virol J. 2018 Mar 22;15:50. doi: 10.1186/s12985-018-0963-1 (PMC5863830; doi:10.1186/s12985-018-0963-1)

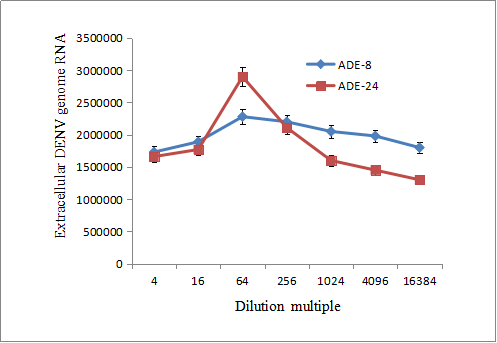

Supplement: Supplementary file 2 — Figure S2. DENV-ADE infection increased virus replication. PBMC cells were infected with DENV3 at a MOI of 5 complexed with 4-fold dilutions of anti-prM mAb. Abscissa expression the dilution multiple of DENV-2 prM antibody, Ordinate representation extracellular DENV genome RNA copy number. (PNG 18 kb) [file 12985_2018_963_MOESM2_ESM.png]

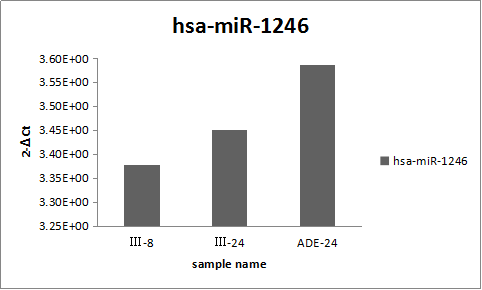

Supplement: Supplementary file 3 — Figure S1. a. Quantitative real-time PCR verify of hsa-miR-1246 among III-8, III-24 and ADE-24. The levels of hsa-miR-1246 is the comparison of PBMC infected with DENV to PBMC infected with DENV-ADE. b. Quantitative real-time PCR verify of hsa-miR-132-3p among III-8, III-24 and ADE-24. The levels of hsa-miR-132-3p is the comparison of PBMC infected with DENV to PBMC infected with DENV-ADE. c. Quantitative real-time PCR verify of hsa-miR-155-5p among III-8, III-24 and ADE-24. The levels of hsa-miR-155-5p is the comparison of PBMC infected with DENV to PBMC infected with DENV-ADE. d. Quantitative real-time PCR verify of hsa-miR-184 among III-8, III-24 and ADE-24. The levels of hsa-miR-184 is the comparison of PBMC infected with DENV to PBMC infected with DENV-ADE. e. Quantitative real-time PCR verify of hsa-let-7e-5p among III-8, III-24 and ADE-24. The levels of hsa-miR-184 is the comparison of PBMC infected with DENV to PBMC infected with DENV-ADE. (ZIP 34 kb) [file 12985_2018_963_MOESM3_ESM.zip › Fig. S1a.png]

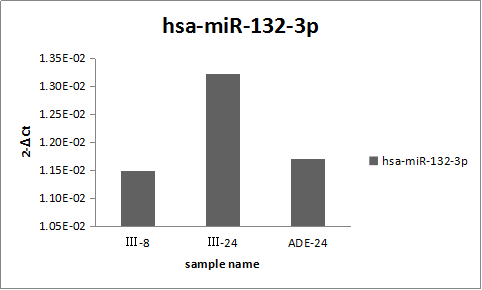

Supplement: Supplementary file 3 — Figure S1. a. Quantitative real-time PCR verify of hsa-miR-1246 among III-8, III-24 and ADE-24. The levels of hsa-miR-1246 is the comparison of PBMC infected with DENV to PBMC infected with DENV-ADE. b. Quantitative real-time PCR verify of hsa-miR-132-3p among III-8, III-24 and ADE-24. The levels of hsa-miR-132-3p is the comparison of PBMC infected with DENV to PBMC infected with DENV-ADE. c. Quantitative real-time PCR verify of hsa-miR-155-5p among III-8, III-24 and ADE-24. The levels of hsa-miR-155-5p is the comparison of PBMC infected with DENV to PBMC infected with DENV-ADE. d. Quantitative real-time PCR verify of hsa-miR-184 among III-8, III-24 and ADE-24. The levels of hsa-miR-184 is the comparison of PBMC infected with DENV to PBMC infected with DENV-ADE. e. Quantitative real-time PCR verify of hsa-let-7e-5p among III-8, III-24 and ADE-24. The levels of hsa-miR-184 is the comparison of PBMC infected with DENV to PBMC infected with DENV-ADE. (ZIP 34 kb) [file 12985_2018_963_MOESM3_ESM.zip › Fig. S1b.png]

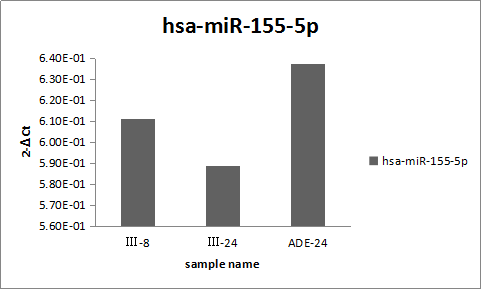

Supplement: Supplementary file 3 — Figure S1. a. Quantitative real-time PCR verify of hsa-miR-1246 among III-8, III-24 and ADE-24. The levels of hsa-miR-1246 is the comparison of PBMC infected with DENV to PBMC infected with DENV-ADE. b. Quantitative real-time PCR verify of hsa-miR-132-3p among III-8, III-24 and ADE-24. The levels of hsa-miR-132-3p is the comparison of PBMC infected with DENV to PBMC infected with DENV-ADE. c. Quantitative real-time PCR verify of hsa-miR-155-5p among III-8, III-24 and ADE-24. The levels of hsa-miR-155-5p is the comparison of PBMC infected with DENV to PBMC infected with DENV-ADE. d. Quantitative real-time PCR verify of hsa-miR-184 among III-8, III-24 and ADE-24. The levels of hsa-miR-184 is the comparison of PBMC infected with DENV to PBMC infected with DENV-ADE. e. Quantitative real-time PCR verify of hsa-let-7e-5p among III-8, III-24 and ADE-24. The levels of hsa-miR-184 is the comparison of PBMC infected with DENV to PBMC infected with DENV-ADE. (ZIP 34 kb) [file 12985_2018_963_MOESM3_ESM.zip › Fig. S1c.png]

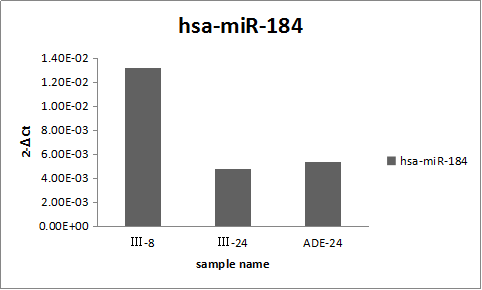

Supplement: Supplementary file 3 — Figure S1. a. Quantitative real-time PCR verify of hsa-miR-1246 among III-8, III-24 and ADE-24. The levels of hsa-miR-1246 is the comparison of PBMC infected with DENV to PBMC infected with DENV-ADE. b. Quantitative real-time PCR verify of hsa-miR-132-3p among III-8, III-24 and ADE-24. The levels of hsa-miR-132-3p is the comparison of PBMC infected with DENV to PBMC infected with DENV-ADE. c. Quantitative real-time PCR verify of hsa-miR-155-5p among III-8, III-24 and ADE-24. The levels of hsa-miR-155-5p is the comparison of PBMC infected with DENV to PBMC infected with DENV-ADE. d. Quantitative real-time PCR verify of hsa-miR-184 among III-8, III-24 and ADE-24. The levels of hsa-miR-184 is the comparison of PBMC infected with DENV to PBMC infected with DENV-ADE. e. Quantitative real-time PCR verify of hsa-let-7e-5p among III-8, III-24 and ADE-24. The levels of hsa-miR-184 is the comparison of PBMC infected with DENV to PBMC infected with DENV-ADE. (ZIP 34 kb) [file 12985_2018_963_MOESM3_ESM.zip › Fig. S1d.png]

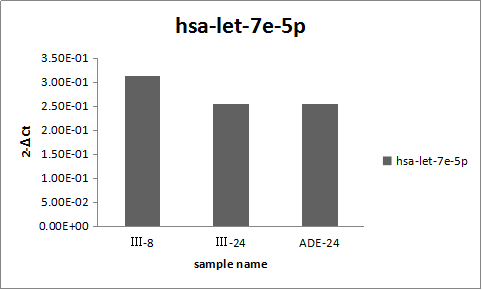

Supplement: Supplementary file 3 — Figure S1. a. Quantitative real-time PCR verify of hsa-miR-1246 among III-8, III-24 and ADE-24. The levels of hsa-miR-1246 is the comparison of PBMC infected with DENV to PBMC infected with DENV-ADE. b. Quantitative real-time PCR verify of hsa-miR-132-3p among III-8, III-24 and ADE-24. The levels of hsa-miR-132-3p is the comparison of PBMC infected with DENV to PBMC infected with DENV-ADE. c. Quantitative real-time PCR verify of hsa-miR-155-5p among III-8, III-24 and ADE-24. The levels of hsa-miR-155-5p is the comparison of PBMC infected with DENV to PBMC infected with DENV-ADE. d. Quantitative real-time PCR verify of hsa-miR-184 among III-8, III-24 and ADE-24. The levels of hsa-miR-184 is the comparison of PBMC infected with DENV to PBMC infected with DENV-ADE. e. Quantitative real-time PCR verify of hsa-let-7e-5p among III-8, III-24 and ADE-24. The levels of hsa-miR-184 is the comparison of PBMC infected with DENV to PBMC infected with DENV-ADE. (ZIP 34 kb) [file 12985_2018_963_MOESM3_ESM.zip › Fig. S1e.png]
